# Bereavement overload and its effects on, and related coping mechanisms of health care providers and ward administrators at National District Hospital in Bloemfontein, Free State

**DOI:** 10.4102/phcfm.v10i1.1652

**Published:** 2018-06-18

**Authors:** Zaid Allie, Edith le Roux, Khantse Mahlatsi, Boitumelo Mofokeng, Zara-Anne Ramoo, Khanyisile Sibiya, Gina Joubert, Jan P. van Rooyen, Hanneke Brits

**Affiliations:** 1 School of Medicine, University of the Free State, South Africa; 2 Department of Biostatistics, University of the Free State, South Africa; 3 Department of Family Medicine, University of the Free State, South Africa

## Abstract

**Background:**

Patient death is an event that all health care workers will face at some point. Beyond the family, the greatest emotional strain is on people who work directly with the patient and family. Bereavement overload occurs after multiple losses without time for normal grief in between.

**Aim:**

To investigate bereavement overload, its effects and related coping mechanisms of personnel working in adult medical wards.

**Setting:**

Four adult medical wards at National District Hospital, Bloemfontein.

**Methods:**

An analytical cross-sectional study design was performed with the aid of an interviewer-administered questionnaire. The target population included health care providers (13 doctors and 20 nurses), eight final-year medical students, and four administrative staff working in the four adult medical wards at National District Hospital, during August to October 2016.

**Results:**

Half (48.9%) of the 45 participants reported bereavement overload. None of the medical students reported bereavement overload compared to 60.0% of nurses, 75.0% of administrative staff and 53.9% of doctors. Nearly two-thirds (64.5%, *n* = 29) stated that they suffered from compassion fatigue. The majority of participants (62.2%) used only positive coping mechanisms. The use of negative coping mechanisms correlated directly with a longer duration in the medical field.

**Conclusion:**

With a 49% prevalence of bereavement overload, it is important that support systems are in place to prevent the effects of negative coping mechanisms. The desirable outcome is that health care providers, who suffer from bereavement overload, experience compassion satisfaction and become more dedicated to the patients’ well-being without expense to themselves.

## Introduction

Psychologist Robert Kastenbaum first described bereavement overload more than 30 years ago as a situation where an individual is grieving more than one loss at the same time, or where losses occur shortly after each other, such that one loss is not dealt with before the other occurs.^1^

Medicine is usually associated with treatment and cure, but there are instances where patients die. Grief is an individual, but normal adjustment process after a loss, such as death.^2^ The grief process affects many aspects of life, including physical, emotional, cognitive, behavioural and spiritual adjustments.^2,3^ Adjustment is therefore needed after each death experience.

In order to adjust, different coping mechanisms can be used. These mechanisms can be either positive, which is behaviour that is beneficial to the person, or negative. Clever et al. first described these coping strategies in their COPE inventory.^4^ These strategies can include seeking support from family or friends, engaging in leisure activities, such as playing with pets, or learning forgiveness and acceptance.

A negative coping mechanism or response is one that does more harm than good. Negative coping mechanisms include behaviour changes, such as alcohol or drug use, isolation and dissociation from one’s reality and avoiding one’s emotions. The long-term effect of negative coping mechanisms, as displayed in burnout and compassion fatigue, negatively affects both the individual and the patient.^5,6^ Maslach conceptualised and defined burnout as: ‘A syndrome of emotional exhaustion, depersonalization, and reduced personal accomplishment that can occur among individuals who do “people work” of some kind’.^7^

Compassion fatigue has been defined as losing one’s ability to care or nurture because of a combination of physical, emotional and spiritual depletion associated with caring for patients in significant emotional pain and physical distress.^5,8^ Compassion fatigue was found to be common among nurses experiencing bereavement overload, and to be proactive, rather than reactive, was the best way to prevent it.^8^

A study conducted in South Africa on failed resuscitations shared the reactions of doctors after the death of a patient. The doctors felt emotionally drained and the incident affected their private lives. Most doctors expressed the desire to have time to reflect on the incident and have time for recreational activities, which was usually not available because of workload.^9^ If another loss will occur before the grief process is completed, bereavement overload will occur.

Support to health care providers is most effective if it was provided in the workplace. Such support should provide a listening ear with simple care and encouragement and not necessarily psychological counselling.^10^ Though the stress and support occur at work, the healing mostly takes place outside the workplace.^11^

Health care providers and hospital staff experience death on a daily basis. They are often seen as strong and expected to move on and continue working on their other patients or tasks with little emphasis on how they deal with the loss personally. Emotional responses are often repressed and may build up or affect the way a person copes later on.

### Aim and objectives

The aim of the study was to evaluate bereavement overload and its effects on, and the related coping mechanisms of, health care providers and ward administrative staff at National District Hospital in Bloemfontein, Free State.

Objectives were:

- To determine the prevalence of bereavement overload.
- To determine the prevalence of compassion fatigue and the relationship with bereavement overload.
- To determine the change in behaviour and attitude towards other patients and self after the deaths of patients, relative to occupation.
- To determine the type of coping mechanisms used after the death of patients.
- To determine if there was a relationship between the occupation, gender and duration in the occupation, and the type of coping mechanisms used.
- To make recommendations regarding coping strategies for students and health care professionals exposed to death and bereavement overload.

## Research methods and design

### Study design, population and sampling strategy

This was an analytical cross-sectional study. The target population included health care providers and ward administrative staff working in the four adult medical wards at National District Hospital in Bloemfontein, during August to October 2016.

National District Hospital is an academic district hospital with 200 beds. There are four adult medical wards (96 beds) where patients are admitted for acute, chronic and palliative care. Because of a lack of other facilities, many terminal and severely ill patients are admitted to the hospital. On average 595 patients are admitted in the medical wards per month and 103 deaths occur. Around 17% of the admitted patients in the adult wards die during their hospitalisation, with an average of three deaths per day.

The target population consisted of all health care providers, final-year medical students and ward administrators of any age and gender who worked in the adult medical wards at National District Hospital in Bloemfontein, Free State, during the study period. These categories of people were all included as they are located in the wards and work directly with patients and their relatives on a daily basis. The population consisted of three consultants, five medical officers, five interns, eight final-year students, 20 nurses and four ward administrators, 45 in total. All members of the target population were included in the sample.

### Data collection

The data for the study were obtained from an interviewer-administered questionnaire that was designed by the researchers after a literature search. The expert advice of two palliative care physicians was incorporated to validate the questionnaire and the questionnaire was piloted prior to use. The questionnaire was quantitative, quantifying the nature of response to bereavement overload and the coping mechanisms thereof, with a few qualitative elements. The questionnaire was available in English as the Free State Department of Health uses English as their main language of communication. The questionnaire consisted of a section for the demographic data of the participants, questions on the death of patients in the wards, bereavement overload, compassion fatigue, coping mechanisms, support structures and the role of education. Participants were reminded of the definitions of bereavement overload and compassion fatigue and it was available in printed form for them to read as well.

The study leader approached each member of the target population and provided them with an information sheet and consent form available in English, Afrikaans and Sesotho. After the participants signed the consent form, their contact details were obtained so that arrangements could be made for the interviews.

Each participant was interviewed by one of the student researchers. The student researchers were in the preclinical phase of study and therefore had no relationship with the participants. The interviews were conducted at National District Hospital. For each of the terms used in the questionnaire, a definition was provided by the researchers (see ‘Definition of concepts’). The presence of bereavement overload and compassion fatigue was self-reported after providing the definitions to the participants. The average length of the interviews was 30 minutes.

### Definition of concepts

Bereavement overload: The grieving of more than one loss at the same time, or shortly after each other, in the work place, in such a way that one loss cannot be dealt with and fully processed before another occurs.

Compassion fatigue: The presence of significant emotional and physical pain or distress causing physical, emotional and spiritual depletion associated with caring for patients.

Coping mechanism: A coping mechanism was regarded as any action or attempt to deal with bereavement overload or patient deaths.

### Pilot study

The pilot study included two medical officers, one intern and one nurse at National District Hospital. The student researchers approached the first person they encountered in any of the wards that were not included in the study in order to test the questionnaire. Some questions were found to be ambiguous and were rephrased, and coding errors were corrected. The results from the pilot study were excluded from the main study.

### Data analysis

The closed-ended questions were coded, and the open-ended questions categorised and coded. All open-ended questions were collectively categorised by the researchers. The symptoms experienced after patient deaths were categorised as; ‘emotional’, ‘physical’, ‘psychological’ or none. Emotional responses included feelings like; ‘depressive’, ‘helplessness’ and ‘out of control’, while psychological responses included ‘irritability’, ‘detachment’ and ‘absent’. Physical symptoms were described as; ‘tired’, ‘sleeplessness’ and ‘body pains’. Responses to the question concerning changes in behaviour or attitude after a patient died were classified in terms of the effect on patients and on self. The nature of the change in behaviour or attitude was categorised according to whether it benefitted (classified as ‘positive’), caused more harm than good (classified as ‘negative’) or was a combination of positive and negative responses (classified as ‘both’).

Categorisation was done in a similar way for coping mechanisms. Positive coping mechanisms included social mechanisms, religious mechanisms and physical activities. Social mechanisms included responses such as; ‘going out’ and ‘talking to family, friends or colleagues’. Religious mechanisms included responses such as ‘praying’ and believing that death was a passage to the afterlife. Physical activities included responses such as ‘gym’. Negative coping mechanisms included doing nothing, detachment and substance use.

The data were entered into a Microsoft Excel spreadsheet. Data analysis were performed by the Department of Biostatistics, Faculty of Health Sciences at the University of the Free State using SAS Version 9.3. Results were summarised by frequencies and percentages. Association between demographic factors and outcomes were assessed using contingency tables with hypothesis testing.

## Ethical considerations

The protocol was approved by the Health Sciences Research Ethics Committee of the University of the Free State (HSREC-S-28/2016) and permission to conduct the study was obtained from the Free State Department of Health. To ensure confidentiality, study numbers were used on the completed questionnaire instead of the participant’s personal information. All participants gave written informed consent.

Arrangements were made with the resident clinical psychologist at National District Hospital in the event that a participant was emotionally affected by some of the questions and needed assistance. During the interviews, the participants were informed of the option to see the psychologist.

## Results

All of the 45 possible participants were interviewed (response rate 100.0%). Two-thirds of the participants were women (68.9%, *n* = 31) and 31.1% (*n* = 14) were men. The highest percentage of participants (44.4%, *n* = 20) were in the age group 21–30 years, 22.2% (*n* = 10) were in the age group 31–40 years and 33.3% (*n* = 15) were older than 40 years. Half (48.9%, *n* = 22) had been in the medical field for 5 or fewer years.

All the participants’ occupations required them to interact with patients and all participants had experienced more than one patient death in the ward. Forty-five percent indicated that they experienced deaths on a daily basis, 51.0% weekly and 4.0% monthly. Only 27.0% of the participants could recall that the death of their first patient elicited a severe emotional response and 62.2% indicated that ‘… it becomes easier …’ to cope with death.

While 48.9% (*n* = 22) of the participants indicated that they are suffering or suffered from bereavement overload, the same percentage (48.9%, *n* = 22) said ‘no’. One participant responded with ‘sometimes’. None of the medical students reported bereavement overload compared to 60% of nurses, 75% of administrative staff and 53.9% of doctors.

Nearly two-thirds (64.5%, *n* = 29) stated that they suffered from compassion fatigue: 50.0% of medical students, 60.0% of nurses, 75.0% of ward administrators and 76.9% of doctors reported this. In the participants with bereavement overload, 77.3% also reported compassion fatigue compared to 50.0 % of those without bereavement overload.

Emotional symptoms because of patient death or bereavement overload were experienced by 62.2%, psychological symptoms by 37.8% and physical symptoms by 13.3%. Nearly a quarter (22.2%) reported no symptoms.

Table 1 shows the change in attitude and behaviour of the different staff members after a patient died in terms of how it affected other patients and how it affected the staff members themselves. Overall, half of the participants (48.9%, *n* = 22) had a positive change in attitude or behaviour towards the patients and 51.1% (*n* = 23) reported a positive change in attitude or behaviour towards themselves. One doctor replied, ‘Sometimes I feel sad, helpless and discouraged, but at the same time more motivated to work harder’. Doctors had the highest percentage of a change in attitude or behaviour that could negatively affect both the patients (46.2%, *n* = 6) and themselves (38.5%, *n* = 5).

**TABLE 1:** The change in attitude and behaviour towards other patients and on the healthcare provider himself or herself after a patient died, grouped according to occupation.

Change in attitude and behaviour after a patient died	Occupation									
	All (*n* =45)		Final-year student (*n* = 8)		Nurse (*n* = 20)		Ward administrator (*n* = 4)		Doctor† (*n* = 13)	
	*n*	%	*n*	%	*n*	%	*n*	%	*n*	%
**Towards other patients**										
No change	11	24.4	1	12.5	8	40.0	0	0	2	15.4
Positive change	22	48.9	5	62.5	9	45.0	4	100.0	4	30.8
Negative change	10	22.2	2	25.0	2	10.0	0	0	6	46.2
Both positive and negative change	2	4.4	0	0.0	1	5.0	0	0	1	7.7
**Towards self**										
No change	11	24.4	0	0	10	50.0	0	0	1	7.7
Positive change	23	51.1	6	75.0	6	30.0	4	100.0	7	53.8
Negative change	8	17.8	1	12.5	2	10.0	0	0	5	38.5
Both positive and negative change	3	6.7	1	12.5	2	10.0	0	0	0	0

† , Includes consultants, medical officers and interns.

As shown in Table 2, 11.1% (*n* = 5) of the participants did not report any coping mechanisms. However, the majority (62.2%) reported using only positive coping mechanisms. Social interaction was the most frequent positive coping mechanism used (48.9%, *n* = 22) ‘detachment’ was the most frequent negative coping mechanism used (22.2%). Examples of the responses were; ‘We discuss it, and then I feel better’ and ‘I try not to think about it’.

**TABLE 2:** Coping mechanisms used by the participants according to occupation.

Variables	Occupation									
	All (*n* = 45)		Final-year student (*n* = 8)		Nurse (*n* = 20)		Ward administrator (*n* = 4)		Doctor† (*n* = 13)	
	*n*	%	*n*	%	*n*	%	*n*	%	*n*	%
**Coping mechanisms**										
Only positive	28	62.2	6	75	12	60	2	50	8	61.5
Only negative	14	31.1	1	12.5	7	35	2	20	4	30.8
Both	3	6.7	1	12.5	1	5	0	0	1	7.7
**Type**‡										
**Positive**										
Social mechanisms	22	22	6	6	8	40	1	25	7	53.8
Religion	9	9	1	1	5	25	2	50	1	7.7
Physical activities	4	4	1	1	0	0	0	0	3	23.1
**Negative**										
Detachment	10	10	1	1	4	20	2	50	3	23.1
Substance use	2	2	1	1	1	5	0	0	0	0
None	5	5	0	0	3	15	0	0	2	15.4

† , Includes consultants, medical officers and interns;

‡ , A participant could indicate more than one coping mechanism.

Most of the final-year medical students (75.0%, *n* = 6), doctors (61.5%, *n* = 8) and nurses (60.0%, *n* = 12) used only positive coping mechanisms. Three-quarters of the final-year medical students (75.0%, *n* = 6) used social interaction to cope, which is higher than the 40.0% and 53.8% reported by the nurses and doctors, respectively. No statistically significant differences were found between the groups.

The majority of participants who had experienced bereavement overload used only positive coping mechanisms (63.6%), as did those who did not experience bereavement overload (59.1%).

Social coping mechanisms were reported more frequently by those who had not experienced bereavement overload (59.1% vs 36.4% of those who had experienced bereavement overload). Religion as coping mechanism was reported more frequently by those who had experienced bereavement overload (27.3% vs 13.6% of those who had not experienced bereavement overload).

The majority of both male (64.3%, *n* = 9) and female participants (61.3%, *n* = 19) used only positive coping mechanisms (Table 3). More female participants (51.6%, *n* = 16) used social interaction compared to male participants (42.9%, *n* = 6), whereas only male participants reported using substances as a coping mechanism. A higher percentage of female participants (25.8%, *n* = 8) used detachment as a coping mechanism compared to 14.3% (*n* = 2) of the male participants. There were; however, no statistically significant differences between gender and type of coping mechanisms used.

**TABLE 3:** Coping mechanisms used by the participants according to gender.

Variable	Years in medical field				*P*-value
	Male (*n* = 14)		Female (*n* = 31)		
	*n*	%	*n*	%	
**Coping mechanisms**					
Only positive	9	64.3	19	61.3	1.000
Only negative	4	28.6	10	32.3	
Both	1	7.1	2	6.5	
**Type**†					
**Positive**					
Social mechanisms	6	42.9	16	51.6	0.7494
Religion	4	28.6	5	16.1	0.4275
Physical activities	2	14.3	2	6.5	0.5776
**Negative**					
Detachment	2	14.3	8	25.8	0.4689
Substance use	2	14.3	0	0	0.0919
None	1	7.1	4	12.9	1.000

† , A participant could indicate more than one coping mechanism.

According to the results in Table 4, 72.7% (*n* = 16) of the participants who have been working ≤ 5 years in the medical field made use of only positive coping mechanisms, compared to 52.2% (*n* = 12) of the participants working ≥ 6 years (*p* = 0.0121).

**TABLE 4:** Coping mechanisms used by the participants according to duration in the medical field.

Variable	Years in medical field				*P*-value
	≤ 5 years (*n* = 22)		≥ 6 years (*n* = 23)		
	*n*	%	*n*	%	
**Coping mechanisms**					
Only positive	16	72.7	12	52.2	0.0121
Only negative	3	13.6	11	47.8	
Both	3	13.6	0	0	
**Type**†					
**Positive**					
Social mechanisms	15	68.2	7	30.4	0.0113
Religion	3	13.6	6	26.1	0.4591
Physical activities	3	13.6	1	4.4	0.3463
**Negative**					
Detachment	4	18.2	6	26.1	0.7222
Substance use	1	4.6	1	4.4	1.000
None	1	4.6	4	17.4	0.3463

† , A participant could indicate more than one coping mechanism.

Participants working ≤ 5 years mostly used social coping mechanisms (68.2%, *n* = 15). Only 30.4% (*n* = 7) of the participants working ≥ 6 years used social mechanisms to cope (*p* = 0.0113). A higher percentage of the participants working ≥ 6 years relied on religious mechanisms compared to the participants working ≤ 5 years (26.1% vs 13.6%).

When the participants were asked if it gets easier to deal with patient death the more they experience it, 62.2% (*n* = 28) said, ‘yes, it becomes easier’ and 37.8% (*n* = 17) said otherwise. Three-quarters of participants felt that their coping mechanisms adequately help them to alleviate emotions or stress from patient deaths or bereavement overload. Most of the participants (68.9%, *n* = 31) reported that they had a support system to deal with the stress and the emotional effects of patient death. Of these, 87.1% (*n* = 27) felt that their support system was effective. Almost all the participants (97.8%, *n* = 44) felt the need for the implementation of education on coping mechanisms and support system in their working environments.

## Discussion

All 45 health care providers and ward administrators in the target population participated in the study. Therefore, the results can be considered representative of the situation at National District Hospital. Half of the participants reported that they suffered from bereavement overload, despite the fact that 96% of them experienced at least one patient death a week. None of the final-year medical students was in this group, possibly because they are in the wards for a limited time of two weeks.

Bereavement overload may lead to compassion fatigue and compromised patient as well as personal care.^6^ Three-quarters of the participants with bereavement overload reported compassion fatigue, meaning that they showed less compassion and caring towards patients. Compassion fatigue was most prevalent in doctors (77%), while 50% of the final-year medical students also reported it. Compassion fatigue may make it easier to cope with the death of a patient, because you spend less time with the patient, are not emotionally so involved and do not know your patient that well. Redingbaugh et al. found that it is harder to cope with the death of a patient who you know well and are emotionally involved with.^12^ Health care providers may use emotional detachment as a mechanism to protect themselves from the impact that a patient’s death may have on them, but this may decrease work satisfaction.

Half of the participants indicated that the death of a patient positively influenced their behaviour and attitude towards other patients and themselves, while in a quarter their behaviour did not change. This positive behaviour change included working harder and being more diligent and empathetic. The literature describes this behaviour as compassion satisfaction and found it protective against compassion fatigue.^5,6,13^

Following the death of a patient, half of the doctors displayed a more negative behaviour in the form of detachment towards their other patients. When a patient dies, doctors attend to the emotions of the family as well as the other team members.^14^ By becoming detached, doctors may find it easier to make decisions regarding their remaining patients, as emotions may cloud judgement.^15^ These behaviour changes were evident in the coping mechanisms employed by the doctors. They either did nothing or became detached. In order to protect themselves against compassion fatigue, they may use perceived negative coping mechanisms such as down-regulation of emotions and empathy to appear professional. A recent study (2014) found that if physicians can down-regulate empathy, they could avoid burnout and compassion fatigue, but still deliver an empathic response towards patients. The correct balance can benefit both the physician and the patient.^16^ Students mainly used social interaction to cope. This can be explained by the fact that students are young and mostly do not have many other responsibilities.

Both genders mostly used only positive coping mechanisms, with no statistical difference between the two groups. As for negative coping mechanisms, more women used emotional coping in the form of detachment, while two men admitted to using more alcohol. These gender differences are supported by literature, where more women were found to use emotional and avoidance strategies while more men as a group used alcohol as a coping mechanism during times of vulnerability.^17,18^ Our study results may not be entirely accurate, as these responses were self-reported, and the numbers are small. Few participants used physical activity as a coping mechanism. Exercise has a variety of health benefits, improves mood and reduces anxiety.^19^

A statistically significantly higher percentage of the group working ≥ 6 years used negative coping mechanisms than those working in the medical field for ≤ 5 years. People working for longer are more likely to be in senior positions and have more responsibility than those with fewer years of work experience. Two-thirds of the participants said that, with time, it became easier to cope with the death of patients.

In the open-ended questions, some of the participants reported that they felt sad, helpless and sometimes discouraged, but at the same time, the experience of patient deaths motivated them to work harder. This positive attitude contributed to compassion satisfaction, which is beneficial to both the health care provider and the patient.^5,6,13,20^

A support system proved to be highly effective for the participants who reported this as a coping mechanism. Many voiced a need for education on coping mechanisms and having support systems in the working environment. Although there is a trained psychologist on site, many of the participants just wanted time to reflect and for someone to listen rather than to seek psychotherapy or counselling.

None of the participants visited the clinical psychologist after the interview process, even though some were very emotional. Members of staff may be reluctant to seek help or do not view the specific emotions as significant enough to be addressed.

### Strengths and limitations

The 100% response rate made the result representative for National District Hospital. The interview process helped to clarify terms like bereavement overload and compassion fatigue; however, it may have contributed to how much sensitive information the participants were willing to disclose. Small numbers hampered comparisons between subgroups. The influence of other factors such as workload, staff morale and limited resources were not investigated as possible reasons for certain responses.

## Conclusion

Half of the participants reported bereavement overload. Compassion fatigue was prevalent in two-thirds of participants and highest in doctors. None of the medical students’ reported bereavement overload, but half did experience compassion fatigue. The majority of health providers with bereavement overload also suffered from compassion fatigue, while half without bereavement overload suffered from compassion fatigue. Compassion fatigue may be a consequence of bereavement overload, and emotional detachment may be used as a coping mechanism.

After experiencing patient death, most participants, regardless of occupation, treated their patients in a more positive way. The sought-after outcome is to have health care providers who are more aware of the potential negative results of bereavement overload after these experiences and become more devoted to the patient’s well-being, without jeopardising their own well-being. The adverse outcome is having health care providers feeling incompetent and worn out and resorting to negative ways of coping such as substance abuse and ignoring the patients’ real needs. Health care providers are doubting their knowledge and are becoming reluctant to treat new patients with similar diagnoses and prognoses as their deceased patients is also an adverse outcome.

Coping mechanisms used by the participants varied, but most used only positive coping mechanisms, such as socialising, religion and physical activities. Different occupations and gender did not influence coping mechanisms. Though detachment was seen as a negative coping mechanism, the down-regulation of emotional responses of doctors showed to be beneficial to both patients and the doctor. The need for education on bereavement overload and the availability of support systems at work was identified.

### Recommendations

The following are recommended:

- Before medical students are exposed to dealing directly with the death of a patient, they should be exposed to the experience of patient death indirectly, for example, an informational DVD or simulation.
- More emphasis should be placed on what to expect when facing a patient death while the future staff member is still in the preclinical phase of their career.
- Thorough and interactive debriefing sessions with a clinical psychologist should be arranged regularly where students can also be screened and scheduled in for an extra session (should they need one).
- Medical staff and students should be encouraged to develop positive coping mechanisms, not only to handle bereavement better but also to cope better with life.
- Staff should be encouraged on a regular basis by those in charge, such as hospital management, to participate in counselling or debriefing or express the need thereof.
